# Effects of an e-learning programme on osteopaths’ back pain attitudes: a mixed methods feasibility study

**DOI:** 10.1186/s40814-021-00901-4

**Published:** 2021-09-13

**Authors:** Jerry Draper-Rodi, Steven Vogel, Annette Bishop

**Affiliations:** 1 University College of Osteopathy, 275 Borough High Street, London, SE1 1JE UK; 2 Arthritis Research UK Primary Care Centre, Research Institute Primary Care Sciences, Keele University, Staffordshire, ST5 5BG UK

**Keywords:** Biopsychosocial, Continuing professional development, Non-specific low back pain, Osteopathy, Manual therapy, Feasibility, Randomised controlled trial

## Abstract

**Background:**

The biopsychosocial model is recommended in the management of non-specific low back pain but musculoskeletal practitioners can lack skills in assessing and managing patients using a biopsychosocial framework. Educational interventions have produced equivocal results. There is a need for an alternative educational tool to support practitioners’ development in the application of biopsychosocial model to manage low back pain.

**Methods:**

A mixed methods study assessed the feasibility and acceptability of an e-learning programme on the biopsychosocial management of non-specific low back pain for osteopaths with more than 15 years’ experience. A sequential explanatory design was conducted, with a feasibility randomised controlled trial and semi-structured interviews explored with thematic analysis.

**Results:**

A total of 45 participants participated in the RCT of which 9 also participated in the interview study. The a-priori sample size was not met (45 instead of 50). The recruitment strategies, randomisation, retention, data collection and outcome measures worked well and were found to be feasible for a main trial. The retention, satisfaction and participants’ views of the programme demonstrated a good acceptability of the programme. Data from the semi-structured interviews were organised in three themes, the first two were related to the feasibility and acceptability of the e-learning programme (practical experience of following the course and engagement with the content) and the third relates to the impact of the intervention (perception of the BPS model).

**Conclusion:**

A main RCT is feasible and the intervention was received well by the participants. A main RCT is required to assess the effectiveness of the e-learning programme. This work also provided data on aspects so far unreported, including osteopaths’ views on continuing professional development, on e-learning as a form of continuing professional development and osteopaths’ perceptions and challenges concerning the implementation of the biopsychosocial model in practice.

**Supplementary Information:**

The online version contains supplementary material available at 10.1186/s40814-021-00901-4.

## Key messages regarding feasibility

- It was uncertain if an e-learning course could be used to train osteopaths to the biopsychosocial model. It was also uncertain if enough osteopaths could be recruited and if the outcome measures would be acceptable.
- The participants accepted well the e-learning course and were satisfied with its content and duration.
- Recruitment strategies and outcome measures could be used in a main trial. The e-learning course was a suitable option as a form of CPD which could be used in a main trial.

## Background

**Fig. 1:**
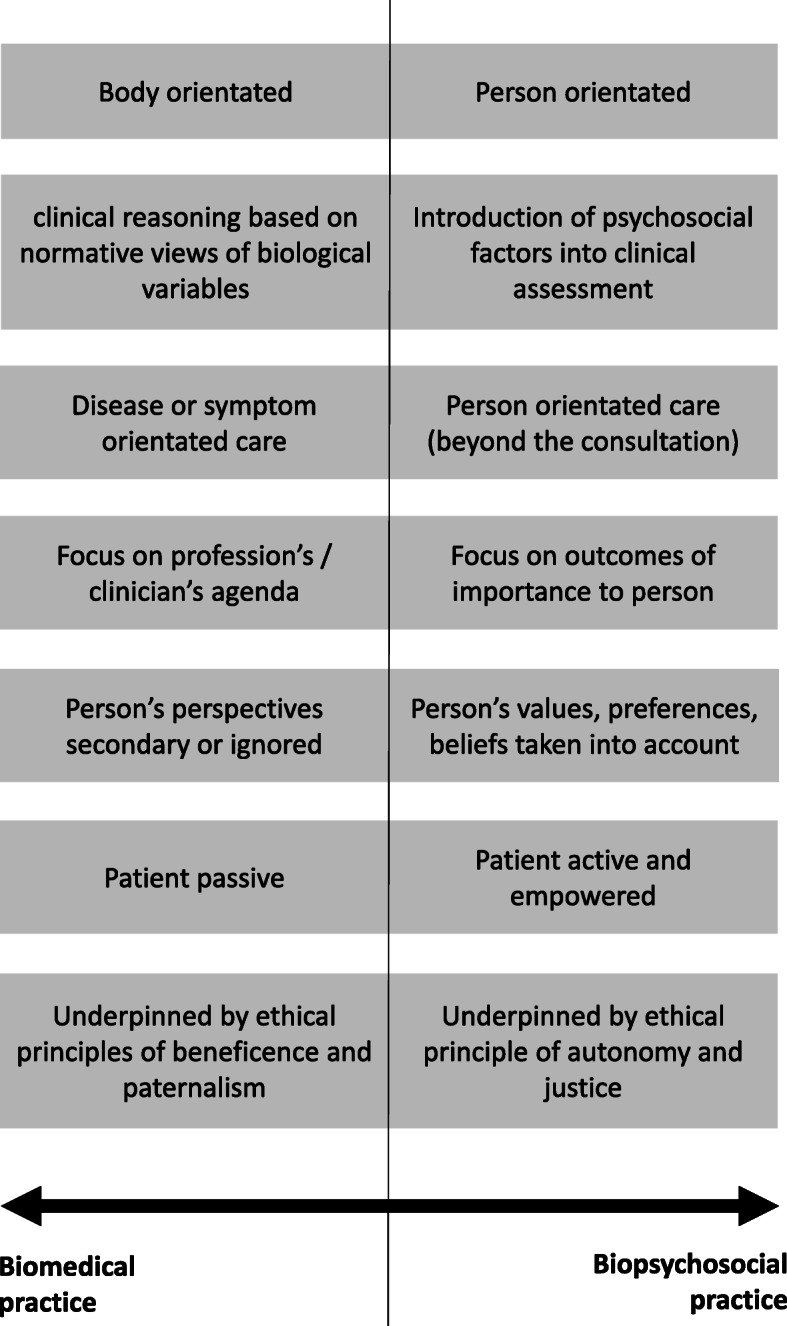
Continuum of practice (the components on the left side of the vertical line correspond to a biomedical style of practice; on the right side to a biopsychosocial one. Note that practitioners may present a mixture of right- and left-side components in their practice resulting in an overall practice style falling somewhere on the continuum). Figure informed by Sacristán [64]

The biopsychosocial (BPS) model has been recommended in the management of non-specific low back pain (NSLBP) for nearly 15 years [12, 54, 55] as NSLBP is multifactorial and BPS factors, such as sleep disorder or depression, are shown to predict pain and disability outcomes [19, 20, 27, 61]. These factors have become targets for intervention [36, 76]. Weighting of factors vary between cases and therefore the expectation is that practitioners are fluent and flexible in their approach in order to most effectively manage patients [13]. This is usually explained as the practitioners’ approach being on a continuum (see Fig. 1 informed by Sacristán [64]). One end being a biomedical orientation is: “a mechanistic view of the body, in which illness is simply a fault in the machine that should be fixed” [81] “and any psychologic element being relatively unimportant or secondary to the physical disorder” [80].

The other end of the continuum being a biopsychosocial one: “a model of human illness (rather than disease) that includes biological, psychological and social dimensions, and the interactions between them” [81].

A practitioner’s orientation can be measured with various instruments, including the Pain Attitudes and Beliefs Scale (PABS) [57] and the Attitudes to Back Pain Scale in Musculoskeletal Practitioners (ABS-mp) [60]. Collectively, manual therapists report a lack of training on BPS assessment and management and express a need for training in this field [24, 68, 71, 82]. Attempts to train manual therapists in the BPS model have had varied results, many with little effect on patient outcomes [29, 38, 58, 69]. Problems identified include interventions being too short (less than 5 h), a lack of needs and content analyses prior to developing the training resource, using small sample sizes (the threshold to observe attitudinal change is 42 participants), absence of explicit use of behavioural change frameworks and poor description of interventions; a common issue with randomised controlled trials [48]. More recent attempts to train practitioners in a BPS approach have been more successful in physiotherapy/physical therapy [2, 4, 31, 59, 70, 78, 79]; however, it remains unclear how best to enhance practitioners’ ability to deliver care using a BPS approach. There is therefore a need to develop and test educational interventions in this field. Osteopaths work mostly independently and tend to be isolated geographically and professionally [23]. Developing CPD to support access to evidence and good practice has been recommended to the regulator of osteopathy [45] and e-learning offers increased accessibility to education, efficacy, cost effectiveness, learner flexibility and interactivity [67], is the fastest growing trend in educational uses of technology [39] and is a mode of delivery that follows good practice advice for medical education [18]. E-learning has not yet been tested for helping manual therapists to use a BPS approach with their patients.

### Aim/objectives

Following the Medical Research Council’s guidance for the development of complex interventions [15], the aim of this study was to assess the feasibility of a main RCT and the acceptability of using an e-learning programme to train osteopaths in the BPS management of NSLBP, who had not been exposed to the BPS model during their undergraduate training. To avoid self-selection bias by osteopaths with a particular interest in BPS, participants were not asked if they had trained in BPS as part of the recruitment process.

Mixed methods were used as recommended in the assessment and/or creation of e-learning programmes in healthcare [10, 62, 77].

## Methods

Guidelines on the conduct of mixed methods research to address processes affecting implementation of evidence-based interventions informed the design of this mixed methods sequential explanatory study [17, 26]. The design was based on a trial with a similar aim, i.e. to assess the effectiveness of a BPS programme on practitioners’ attitudes [59]. The research was approved by the University Research Ethics Committee. The trial was not registered.

### Quantitative strand

This is reported in line with the CONSORT guidelines [49].

#### Trial design

**Fig. 2:**
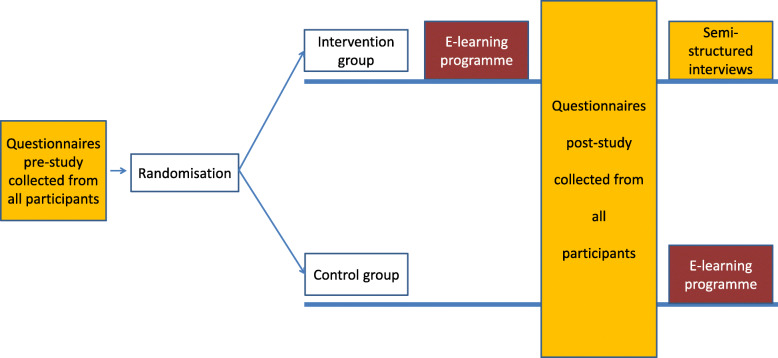
Study design

The study was a feasibility RCT with a parallel design (see Fig. 2) to control for confounding factors external to the study. The allocation ratio between the intervention group and the control group was 1:1. There were no important changes to the methods after the trial started.

#### Participants

To be eligible for the study, participants had to:

- Be an osteopath practising in the UK
- Have a minimum of 15 years’ practice experience; undergraduate curricula have integrated BPS principles in recent years but experienced osteopaths would not have been trained on this model during their undergraduate training
- Not have been involved in osteopathic education in the last 10 years

Those eligible and agreeing to take part provided written consent.

#### Recruitment

The 6-week recruitment period started on 01/09/2015 accessing a national sampling frame via a number of different recruitment strategies. Emails to osteopaths randomly selected from the General Osteopathic Council database of those indicating availability to be contacted for research purposes, and additional direct contact was made with regional groups. The National Council for Osteopathic Research disseminated information about the opportunity to take part through social media and regional research hubs were also invited to alert their members. Adverts in professional journals also appeared. The wording chosen presented the material in a factual manner in order to avoid self-selection bias by those with a particular interest in BPS. Prospective participants who expressed an interest contacted the principal investigator who sent a participant information sheet and consent form in an email approved by the research ethics committee. No financial incentives were offered to take part.

#### Intervention

**Fig. 3:**
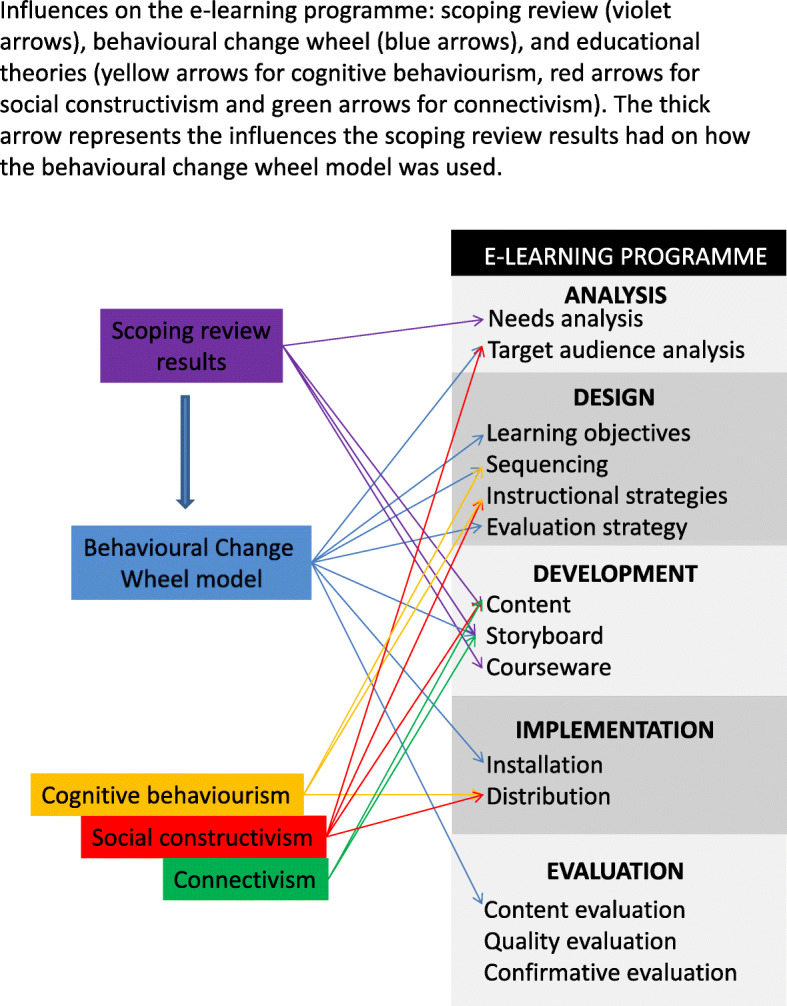
Intervention development

The intervention was developed for participants who had significant clinical experience but more limited exposure to contemporary evidence and little exposure to the concepts underpinning the BPS model. This had been explored qualitatively prior to the development of the e-learning programme and a need had been found to close the theory-practice gap, requiring specific training to change manual therapists’ attitudes to back pain, knowledge, skills and confidence to assess and manage patients within a BPS framework. Findings were similar across different manual therapy professions [24, 82]. The e-learning programme for this study was developed by an osteopath (JDR) and the content was audited by two BPS experts: an osteopath (SV) and a physiotherapist (AB). Its development is detailed elsewhere [19, 20] but, in summary, it involved applied theories that informed different stages (see Fig. 3). The theoretical underpinning included results from a scoping review [19, 20], the behavioural change model [47] and educational theories; and the e-learning was arranged following the ADDIE stages model of e-learning programmes.

**Fig. 4:**
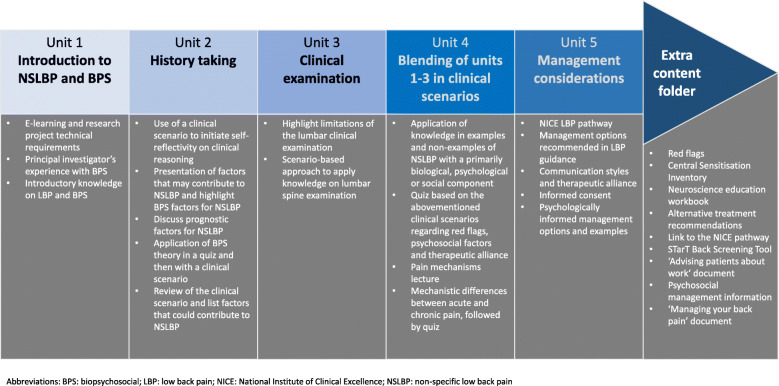
E-learning content (the arrow follows the sequencing of the units. The units were instructor-led)

The e-learning was developed using a Moodle platform and included lectures, interactive case scenarios and quizzes. Its duration was 8 h informed by the ADDIE development phase. Each lecture was maximum 30 min to enhance participants’ experience. There was no formal interaction between the research team and the participants. Participants could, however, email the research team if needed. It was organised into 5 units (see Fig. 4): unit 1 provided general information on NSLBP and the BPS model; unit 2 focused on history-taking; unit 3 on clinical examination; unit 4 integrated the content of the previous units using clinical scenarios and unit 5 discussed broader management considerations. Access to each unit was granted once the previous unit had been completed (the content was not graded but participants had the option to retake quizzes after reading feedback on their answers). The e-learning also included an extra-content material section where participants could access resources and materials to further their knowledge on a topic.

All participants were informed that the course would require a total of 8 h over 6 weeks. The intervention group was invited to take the e-learning programme on 19/10/2015, whilst the control group participants were informed they had to wait for the other group to complete the e-learning programme and their starting date, 06/12/2015. They were managing their patients as usual during that time. They all had 6 weeks to complete the e-learning programme. Participants’ engagement with the module was monitored once a week using the e-learning programme administration panel on the e-learning programme website. Participants were contacted by email after seven consecutive days without logging in, over the phone after 14 days, and by text message after 21 days.

#### Data collection

At baseline, all participants were asked to complete the initial questionnaire that included participant characteristics and two validated attitudinal measures (not explicitly named in the questionnaire): the Pain Attitudes and Beliefs Scale (PABS) [57] and the Attitudes to Back Pain Scale in Musculoskeletal Practitioners (ABS-mp) [60] that have acceptable psychometric properties [5, 7, 8, 32, 33, 52, 57, 75] (see Table 1).

**Table 1:** ABS-mp and PABS subscales (the last column is specific to this research project which aimed to change participants’ attitudes to back pain)

Scale name	Subitems	Subitem detail	Score	Predicted direction of change in intervention arm
ABS-mp	Personal interaction consists of four factors			
	LS	Limitations on sessions, items about practitioners’ policy towards limiting the length of treatment (four items).	28	Unknown
	PS	Psychological, items measuring practitioners’ willingness to engage with psychological issues with their patients (four items).	28	Unknown
	CHS	Connection to healthcare system, items measuring practitioners’ perception of the health-care system and provision of available services (three items).	21	Unknown
	CC	Confidence and concern, items measuring practitioners’ confidence and concern about treatment and clinical limitations in themselves and others (two items).	14	Unknown
	Treatment orientation consists of two factors			
	RA	Re-activation, items that concern return to work and to daily activity and increasing mobility (three items).	21	↑
	BM	Biomedical; items that concern advice to restrict activities and to be vigilant, and the belief that there is an underlying structural cause of back pain (3 items).	21	↓
PABS	Biomedical	Practitioner believes in a biomechanical model of disease, where disability and pain are consequences of specific tissue pathology and treatment is aimed at treating the pathology	10	↓
	Behavioural	Practitioner believes in a biopsychosocial model of disease, in which pain does not have to be a sign of tissue damage and can be influenced by social and psychological factors	9	↑

Unknown: items that may be influenced by a biopsychosocial training intervention but direction of change currently unknown

Participants were invited post intervention, to complete a follow-up questionnaire which included the attitudinal measures and a short satisfaction survey on the e-learning programme assessing their satisfaction with the e-learning programme, their interest in the e-learning programme, new perspectives on NSLBP and the clarity of teaching of the e-learning programme.

#### Sample size

Following guidance on participant numbers for feasibility studies [14, 42] and on how feasibility RCTs can provide reliable standard deviation estimates for a power calculation [66], a total sample of 50 participants was sought for inclusion in the feasibility RCT.

#### Randomisation

The randomisation procedure was implemented by the unblinded principal investigator using the RAND function in Excel which generated a random number sequence used to allocate participants to groups.

#### Blinding

Participants and the researcher who collected and analysed the data were not blinded to group allocation. This research was part of doctoral work and the PI conducted all the different stages of the work to gain experience in different aspects of trial research.

#### Statistical methods

All statistical analyses were performed using IBM SPSS version 22 (IBM Corp, Armonk, New York). Being a feasibility study, the analysis was descriptive and focused on mean difference and 95% confidence intervals and not on inferential testing [41–43, 50, 73]. The survey data were summarised using medians, interquartile ranges and percentages. An open text question asking about the ‘Three most useful things learnt’ was analysed using content analysis [28] where items were counted to list and rank the participants’ views on the most useful things learnt. Frequencies reported the number of individual participants who mentioned a particular theme, rather than the number of times themes were mentioned, to prevent over representation of individual participants who could mention a theme several times [53].

### Qualitative strand

Semi-structured interviews were used to collect more in-depth views and opinions on the e-learning programme from a convenience sample drawn from the intervention group.

#### Participants

All participants from the intervention group were sent an invitation inviting them to take part in an interview conducted using a voice-over-IP service (such as Skype®) with video feature based on participants’ previous experience and preferences. Before recording, the consent form content was discussed, participants were reminded they could withdraw at any time without needing to give reasons, and to keep the interview content confidential. Participants were informed that a device was used to record the interview and when it was turned on. At the end of the interview, the participant was thanked and offered the opportunity to review and amend the transcript before it was used in the analysis. The researcher stopped the online conversation.

#### Data collection and analysis

An interview guide was used during the interviews to gather participants’ views on the intervention itself (see Table 2) and to explore if and how the e-learning intervention had an impact on their practice. The interview was transcribed using a six-step reflexive, iterative process of data management [28] and analysed with both content and thematic analyses ([9, 16, 53], p. 251 and 433, [34]). The data were coded identifying themes or patterns. Themes were then reviewed and refined [9] in order to identify key themes, areas of consensus and differences of opinion between participants. Data triangulation was used to assess saturation ([17], p. 251 and 433, [34]). Audiotapes were used to identify illustrative quotes to illustrate themes.

**Table 2:**
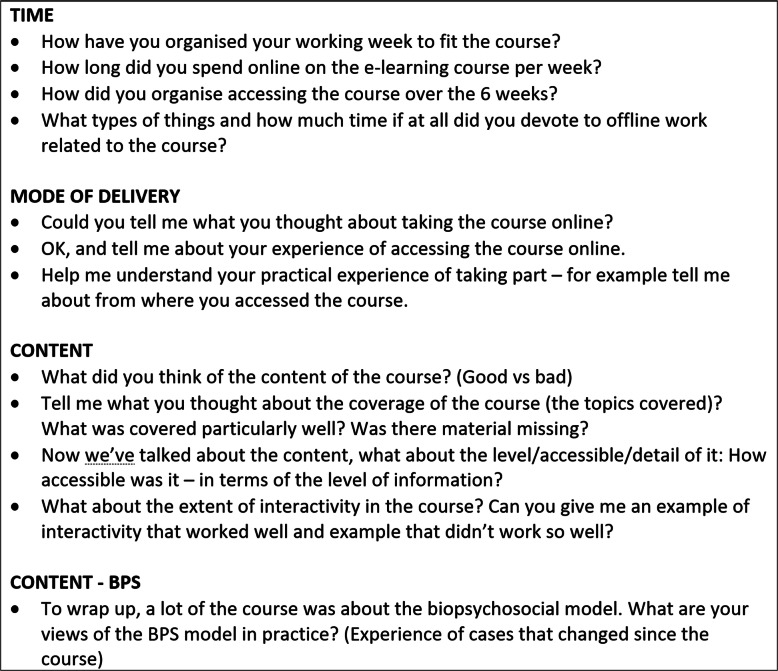
Interview guide

## Results

**Fig. 5:**
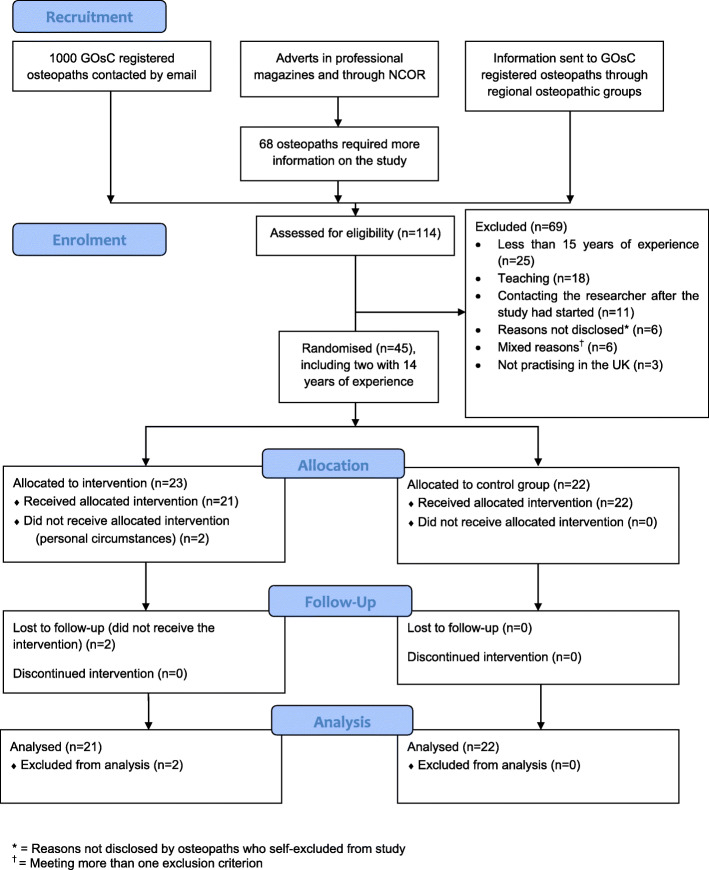
Study flowchart

A total of 45 participants took part in the feasibility RCT: 23 were randomly allocated to the intervention group and 22 to the control group. The demographics of the participants in both groups are presented in Table 3—the main difference between the groups was their special interest in LBP: the intervention group had twice as many as the control group. Recruitment and participant flow are reported in Fig. 5.

**Table 3:** RCT participants' characteristics

	Intervention group (*n*=23)	Control group (*n*=22)
Female n (%)		
Age group (number of participants)	30-39 (*n*=1)	30-39 (*n*=2)
	40-49 (*n*=9)	40-49 (*n*=9)
	50-59 (*n*=12)	50-59 (*n*=7)
	60-69 (*n*=1)	60-69 (*n*=4)
Median (IQR)	4.00^a^(1.00)^b^	3.50 (1.00)
	(50-59)	(40-49)
Years in practice Mean (SD)	21.91 (5.74)	23.45 (5.26)
Special interest in LBP n (%)	14 (61%)	6 (27%)

^a^ 4 is 50-59 age group; ^b^ Quartile 3 is 40-49, quartile 1 is 30-39

### Qualitative strand

Nine participants from the intervention group took part in the semi-structured interviews. The participants’ demographics are shown in Table 4.

**Table 4:** Semi-structured interview participants’ characteristics

ID	Gender	Age group	Years in practice	Special interest in LBP
107705	Female	50-59	26	Yes
117268	Male	40-49	25	Yes
215827	Female	50-59	23	Yes
375469	Male	50-59	29	No
410737	Female	40-49	17	Yes
431276	Male	60-69	31	Yes
532034	Male	50-59	17	Yes
539532	Female	40-49	18	No
878115	Female	40-49	23	Yes

### Feasibility of a main trial

To assess the feasibility of a main trial, Table 5 describes the integrity of the study protocol; specifically, the feasibility of the recruitment strategies, the recruitment and retention rates, the randomisation procedure, data collection and outcome measures. This mixed methods study followed the protocol that would be followed for a larger trial, including inclusion/exclusion criteria, and intervention preparation and testing.

**Table 5:** Feasibility criteria

	A priori feasibility criteria	Feasibility threshold met?	Considerations for future trial
Recruitment strategies feasibility	Journals agreeing to publish the recruitment ad for free (^a^)	Both professional journals accepted	It is not possible to provide an accurate recruitment rate as some of the advertising media contacted osteopaths indirectly and so the denominator is unknown.
	Regional groups accepting to forward messages to their members	All regional groups accepted	
	National Council for Osteopathic Research (NCOR) accepting to inform their members about the research project (^a^)	NCOR informed their members and mentioned the study when doing talks to regional groups	
	Ads published on time (^a^)	Journal ads were published on time for readers to have time to contact the PI if interested to have more information	
	Gaining access to GOsC members’ email addresses who accepted to be contacted for research purpose (^a^)	Access to 1000 email addresses was granted by the PI’s institution	
	Mail Merge system allowing to do mass emailing (^a^)	1000 emails were sent with no issue with the system	
Recruitment and retention feasibility	Recruiting 50 participants (^a^)	Only 45 were recruited. As only 43 met the eligibility criteria, two with 14 years in practice were included.	• Reassess eligibility criteria owing to their strictness • Consider recruiting other Allied Health Practitioners with similar scope of practice • Consider having a longer recruitment period (12 prospects contacted the PI within the following 4 months).
	Enough participants accepting to be interviewed to reach data saturation (^b^)	10 participants agreed to be interviewed, 9 were needed to reach data saturation.	
	Participants sending back consent forms (^a^, ^b^)	Consent forms were received from all participants	
	Reaching an 80% retention rate (^a^, ^b^)	91% (41/45) for the Quant strand, and 90% for Qual strand (9/10)	High retention rate achieved by sending reminders to participants: up to seven reminders per participant when not accessing the e-learning
Data collection feasibility	Receiving questionnaires back from participants (^a^)	Post-intervention questionnaires were completed by 43 participants (96%): 2 participants in intervention group did not complete the course (details in text below) nor questionnaires despite invitation. All control group participants completed the questionnaires	
	To send questionnaires in a format easy to access and fill in by all participants (^a^)	Questionnaires sent in Word© were protected to ensure participants could not change the content of the questionnaires. This led to compatibility problems.	Alternative systems should be considered.
	Using voice-over-IP service successfully (^b^)	Yes	
	Being able to record the interviews (^b^)	One recording was faulty. Field notes were used to write up a transcript that the interviewee checked within three hours of the interview. No excerpts were used from this interview.	
	Interview guide suitable (^b^)	Provided useful content and was well accepted by participants	
	Participants agreeing to fill in questionnaires (^a^)	Participants accepted the questionnaires well. Some were surprised about the similarities of the questions across the two validated measures included.	Consider using only one of the two attitudinal questionnaires.
	Control group accepting to fill in questionnaires twice before taking the course, 6 weeks apart (^a^)	All sent back the completed questionnaires	
	Suitability of the reflexive iterative process of data management (^b^)	The process was feasible and satisfactory and was well accepted.	
Acceptability and feasibility of the outcome measures	Suitability of attitudinal questionnaires (^a^)	Some participants expressed their surprise about the similarities of the questions across the two validated measures included	
Threshold met	Threshold partly met	Threshold not met	

^a^ Quant strand, ^b^ Qual strand

### Feasibility and acceptability of the e-learning programme

The feasibility and acceptability of the e-learning programme are presented using the satisfaction survey results and the participants’ views on the e-learning programme.

#### Satisfaction survey

**Fig. 6:**
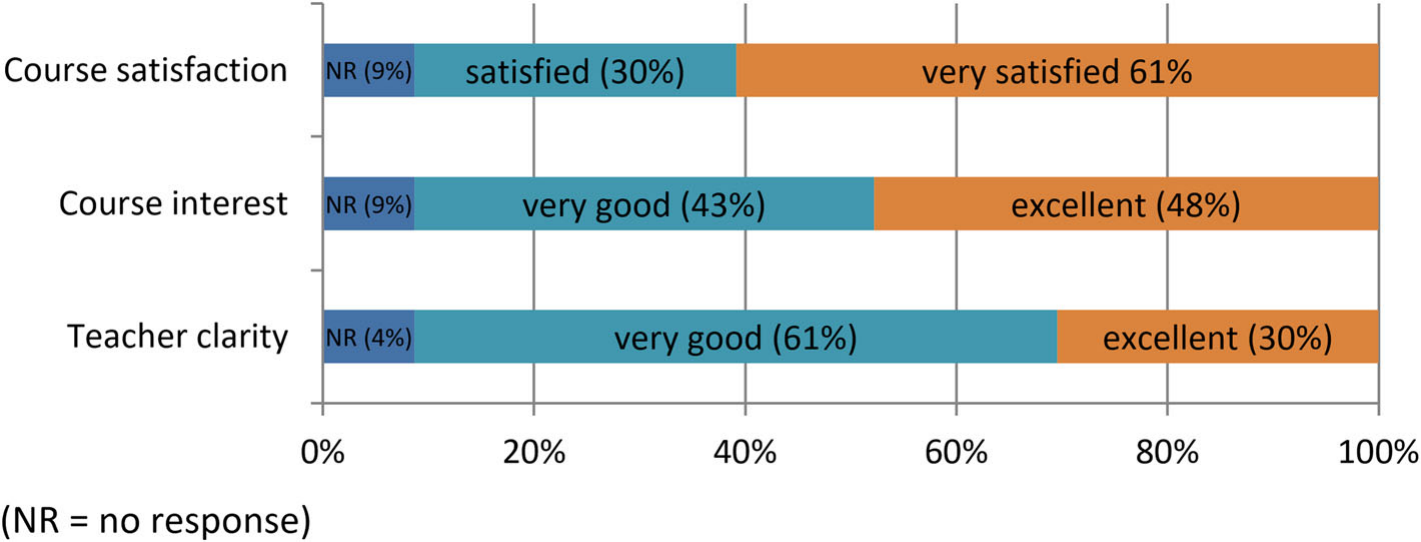
Answers to satisfaction questions

Twenty-one out of 23 participants from the intervention group answered the survey at the end of the e-learning programme. The responses to the satisfaction questions are summarised in Fig. 6, which show high levels of satisfaction. No participant rated the course as unsatisfactory, teacher clarity or course interest as less than very good.

All but one participant (*n* = 20) responded to the question stating the three most useful things learnt during the e-learning programme. Content analysis suggested 3 categories: answers related to pain theory (21), to patient management (18) and to BPS influences and diagnosis (18).

#### Participants’ views on the programme

Data from the semi-structured interviews were organised in three themes. The first two themes are presented in Table 6, *Practical experience of following the course* and *Perception of the content*, and the third follows in the ‘Impact of the e-learning programme’ section, which uses both quantitative and qualitative data*.*

**Table 6:** Qualitative views on the e-learning programme (first two themes from semi-structured interviews presented in this table, third presented in-text below)

Subtheme	Summary description	Illustrative quotation
**Theme 1: Practical experience of following the course**		
Time and setting	Time outside clinic time was favoured to not disturb clinic schedule. Weekday evenings were the most popular time. The course was mostly completed in chunks, at their own pace.	“It worked better for me doing it in chunks” Participant B
Practical aspects of the course	The mode of delivery of the course was well accepted by the participants. They were happy that it was online.	“It was very good, very convenient in the sense that I could do it from home or in my clinic if I wanted to. I didn’t have to travel to a venue.” Participant G
	The e-learning programme was described as easy to access, including in areas with low broadband connection or on different operating systems. Some minor difficulties were reported (e.g. slight confusion during initial connection for one participant; e-learning website down one half day when the hosting server was down; and one participant found not very clear how many lessons had been completed). The e-learning programme was described as well-presented and very easy to access from a laptop. One interviewee reported that the references font was too small to be read on tablets. The interactivity was thought to work well and the use of quizzes was particularly praised.	“Quizzes made me think and made me have to recall what I’d been looking at and listening to so I thought they were really good. They helped to reinforce the learning.” Participant D
**Theme 2: Perception of the content**		
Engagement with the content	Participants found there was sufficient content, the e-learning programme was thorough whilst not being overwhelming and being accessible to the participants with little academic background.	“A lot of content and it was very relevant to clinical you know to practice of osteopathy and the type of patients one sees. And you explained the concepts very well which being an older practitioner, someone that qualified a long time ago I guess a lot of these concepts were not around then so it was good to have your programme there which introduced a lot of these concepts in a very clear manner” Participant G
	Participants were satisfied with the content and the coverage of the e-learning programme and found the content clear.	“There were things within the content that I’d certainly come across like the flags etc. before on other courses but actually it was a much more interesting approach to the flags that I’ve come across before” Participant I
	Quizzes were found helpful to engage with the content and made participants study and reflect. They helped the absorption of the information delivered. Seeing results was found gratifying.Consideration for quiz improvements: providing a record of previous attempts when participants take quizzes multiple times; distributing them more across the e-learning programme (e.g. Unit 5: management considerations had no quizzes); and using more learners’ experience in the questions.	“The last quarter [of the e-learning programme] I thought “Gosh there’s a lot there” in the last chunk which I felt was clinically very relevant” Participant G
Extra work done	Nearly half of participants interviewed spent more than the estimated 6 h taking the course (range from 8 to 12 h).	“I did go back to a couple of the modules just to understand them again. I did a couple of modules further on and I thought “oh I think I need to go back to the basics” so I went back to the earlier modules a couple of times” Participant E
	The extra work also included the ‘Extra content’ folder, accessed by all but one of the participants. It also included taking notes and reading them back or taking screen pictures as memos and looking at them later. One participant mentioned that a handout would have been useful.	“I estimate eleven hours [of online work]: I went back over bits; I made notes when I went along as well” Participant B

### Impact of the e-learning programme

#### Participants’ perceptions of the BPS model

The final theme emerging from the interview data characterised study participants’ perceptions of the BPS model and was formed of the three sub-themes *BPS model is not structural enough*, *BPS model is part of existing practice*, and *Transformative*. These perceptions are presented below with illustrative quotations. This is not to suggest that all participants fitted distinctly in each sub-theme (as there was some overlap) but rather it offers a broad, differentiation of participants’ perceptions.

#### BPS model is not structural enough

One view of the BPS model was that it was not sufficiently based on biomechanical and anatomically focussed care (aka as structural approach). It was perceived as a model where musculoskeletal problems were either systemic (red flags) or psychosocial (yellow, blue and black flags) with no space for simple mechanical aetiology.

"There is a psychological element to it, there’s a social element to it but there’s also possibilities of physical problems which are not pathological but are not psychological or social". Participant A

This lack of a structural aspect led to a sense that osteopathy was devalued in the content of the e-learning programme.

"There was a general tone of - I would quite often - you know this thing about I’m going to go and shoot myself now then - what am I doing as an osteopath? You know? There was a general tone of devaluing what osteopaths do". Participant A

#### BPS model is part of existing practice

The BPS model was viewed as a model of practice that was already used and familiar to osteopaths.

"I think intuitively a lot of osteopaths do follow some of the concepts [of the BPS model]" Participant G

Whilst there were no disagreements with the content, there was a feeling that the content was not bringing a new perspective to osteopaths on back pain.

"Some of the psychological and psychosocial stuff I think a lot of older people that are reasonably experienced, I think we do it anyway" Participant A

#### Transformative

The last category saw the BPS model as a better model than the biomedical, and one that was suitable a meta framework for practice.

"That [BPS] model works better for me than the biomedical one which actually has always been a bit of a struggle to know ‘is it facet?’ ‘is it disc?’" Participant I

The BPS model was seen as offering a novel approach to back pain. It had not been taught during undergraduate education despite experience of subjects such as psychology or diet. Participants described isolated topics presented in a mechanistic nature and not integrated in clinical practice.

"That’s something I wasn’t taught a great deal at undergraduate when I was a student although I’ve heard about it postgradutately (sic). The Flags were new to me so I found that very helpful". Participant B

"[The content] was very good, very thorough. It was an aspect of diagnosis I hadn’t learned in college so it did make me think. It challenged the way I had been taught" Participant E

It also had the merit of being evidence-based rather than experience-based.

"It was very helpful, it was drawing on research because so much we’re told, or what I was told in my training was basically experiential" Participant H

The BPS model offered a structure to assess and manage patients with, e.g. the flag system and a system to integrate the different aspects of a patient’s life. It also helped patients’ management.

"It has made me think a bit more about the various factors which do come to play in a person’s problems which would stop them getting better. Since doing the course I have identified people who had put perhaps psychological barriers up to their progress or to advice on exercises". Participant E

Participants became more aware of the risks of increasing patients’ negative attitudes to back pain. To prevent this, participants changed their communication content and style with patients.

"[The course] has changed in some of the language maybe that I would use with patients and just re-emphasizing thought positives and maybe not using quite so much medicalised language". Participant I

The BPS model also offered a common language with other professionals.

"It seems to be absolutely everywhere at the moment. It seems to be the way the NHS is going in this country, the way physios are going in this country so I think it’s something we need to embrace - that we need to be very aware of". Participant I

### Questionnaire data

There was little difference in the means and standard deviations on the six ABS-mp domains and the two PABS domains for the intervention and control groups at baseline.

Between-group changes on the ABS-mp show that 3 domains had mean differences with confidence interval ranges that did not include the value of no effect: LS, PS and BM; and on the PABS both domains had mean differences with confidence interval ranges that did not include the value of no effect.

Table 7 details within group and between group changes in attitudinal measures.

**Table 7:** Attitudinal questionnaires results (within-group and between-group)

		Baseline	After	Mean difference within group	95% confidence interval of the difference		Mean differences between groups	95% confidence interval of the difference	
					Lower	Upper		Lower	Upper
ABS-mp	LS	17.4	13.8	3.6	1.8	5.4	2.2	0.1	4.3
	LS	18.4	17.0	1.4	0.1	2.7			
	PS	20.5	22.6	−2.1	−3.1	−1.1	−2.2	−3.5	−0.9
	PS	20.7	20.6	0.9	−0.7	0.9			
	CHS	10.0	9.6	0.4	−1.0	1.7	0.4	−1.2	2.0
	CHS	11.6	11.6	0	−1.0	1.0			
	CC	8.3	8.7	−0.3	−1.4	0.7	−0.8	−2.1	0.4
	CC	9.4	8.9	0.5	−0.2	1.2			
	RA	14.8	16.3	−1.6	−2.8	−0.3	−0.8	−2.4	0.9
	RA	14.2	15.1	−0.8	−0.2	0.3			
	BM	13.5	9.3	4.2	3.1	5.4	4.1	2.8	5.4
	BM	13.6	13.4	0.2	−0.6	0.9			
PABS	Biomedical	35.3	25.7	9.6	7.6	11.7	11.0	8.2	13.8
	Biomedical	34.8	36.2	−1.4	−3.4	0.6			
	Behavioural	29.9	35.0	−5.1	−7.4	−2.9	−3.5	−6.3	−0.6
	Behavioural	29.6	31.2	−1.7	−3.5	0.2			

Intervention group (n = 23)

Control group (n = 22)

## Discussion

This feasibility study found that overall using an e-learning programme to train experienced osteopaths to the BPS model regarding NSLBP was acceptable and feasible in all points, except for the recruitment as the a-priori number of participants was not reached.

### Feasibility of a main trial

#### Recruitment

The recruitment strategy included the use of several different media to assess if this could provide enough participants in a main trial. It was found that all media were satisfactory; however, further more collaborative efforts with copy editors are recommended to ensure that the material published is fully aligned with the required copy provided by the research team. Using phone calls or sending SMS could complement the recruitment strategies used as they are effective ways to increase recruitment rates [74] but careful consideration would need to be taken regarding practical, ethical and resource implications. Being a feasibility study, 50 participants were sought for the mixed methods feasibility study. Only 45 were recruited and this was largely due to the strict exclusion criteria. One exclusion criterion was to have not taught in the previous 10 years. This was based on a supposition that educators could have been exposed to the BPS model during their teaching. A recent qualitative study conducted in New Zealand [63] analysed video-recordings of the clinical management of patients with acute NSLBP by 3 osteopaths who graduated in the UK and were teaching in New Zealand. The model used by these participants includes clear signs of BPS management supporting the exclusion criterion choice. Another possible reason for the low recruitment rate might have been related to the recruitment period: 12 participants contacted the researcher after the study had started, up to 4 months later. For a further study, it would be recommended to extend the 2-month recruitment period and to carefully consider the exclusion criteria for recruiting more participants whilst weighing the risk of having a population that would not respond to the intervention. Another way to improve recruitment would be to enhance the description of the e-learning programme by including the possible effects of the e-learning programme on clinical practice and the individual benefits for participants [22]. Low recruitment rates in trials are a common problem with less than a third achieving the recruitment of the number of participants initially planned [72]. A possible way forward for a further study would be to include practitioners from different manual therapy professions.

#### Randomisation and data collection

The randomisation process, using the RAND function in Excel, worked well and could be employed in a main trial. Baseline characteristics were balanced apart from the special interests between the groups. This may be due to the small sample size in this feasibility study and in a larger main RCT all baseline characteristics should be balanced. If baseline characteristics imbalance remains, it may be associated with the outcome [11] and should be accounted for in the analysis [21]. The retention rate was high, potentially due to having a highly selected sample. The impact on retention would need to be considered if the inclusion criteria were changed for a main trial. Using digital versions of the questionnaires to collect participants’ answers was found adequate. Few participants decided to send hard copies.

#### Outcome measures

In the main trial, PABS on its own could be used as both questionnaires showed similar changes and the PABS permits comparing findings with other studies.

### E-learning acceptability

The acceptability of both the content and the instructional method was overall good.

Participants valued the Extra Content Folder; the fact that the content was evidence-based rather than experiential and that references were listed. One participant mentioned in their semi-structured interview a need for an easier system to contact the lecturer than email. As participants valued the autonomy they had whilst taking the course over the 6-week period with no constraints on time, place, or from other participants, asynchronous collaboration and communication tools (e.g. emails and forums) would therefore probably be easier and more appropriate to implement than synchronous tools (e.g. live instant messages and live broadcasts) in an improved version of the e-learning programme. This would also be better educational tools as using those asynchronous format in e-learning programmes for postgraduate studies promotes self-reflection [67] leading to deeper learning than e-learning programmes using a synchronous format [46].

#### Content acceptability

The content satisfaction was high due partly to the content being evidence-based rather than experience-based or anecdotal which provided participants with clear tools and approaches to discuss management options with patients (e.g. the possible innocuousness of some MRI findings). This was also reported in another study that assessed what participants found helpful to change their attitudes to back pain [56].

Whilst participants generally reported that nothing was missing from the e-learning content, there was overall agreement that more information on how to implement a BPS management of patients with NSLBP was required (developed in Unit 5 of the e-learning programme). Participants suggested that this information should be developed in a different e-learning programme, as the one developed in this study already contained a lot of content.

#### E-learning programme impact and contextualisation

There is currently no definition of what constitutes a high or low score on the ABS-mp or PABS domains, making it difficult to quantify a clinically relevant attitudinal change [52] but results from our mixed methods feasibility study were consistent with scores found in previous studies that also used the PABS [6, 25, 30, 32, 33, 35, 59]. Participants in this study had slightly higher biomedical scores and lower behavioural scores than Houben et al.’s participants (2005, 2005). Participants included in the study were experienced osteopaths whereas Houben et al.’s participants were either physiotherapy students or physiotherapists with an average of 12 years of work experience. This is consistent with results in a study that found that the more experienced GPs are, the more likely they are to have high biomedical levels [25].

The intervention group in our study showed changes in scores on the PABS domains after an 8-h e-learning programme: the biomedical score decreased and the behavioural score increased. Those changes were significant when compared to two studies that also used PABS as their outcome measurement questionnaire [4, 59]. The effect size of the intervention was large on both PABS subscales (2.4 for the biomedical subscale and 0.75 for the behaviour) but caution would be required if these findings were used in a main trial due to limitations in using feasibility studies to provide precise between treatment group effect size estimates [1, 14, 40, 43].

### Strengths and limitations

This feasibility study was the first to assess osteopaths’ views on using e-learning as a form of CPD and their views on the BPS model. The design followed best practice: the MRC’s recommendations for the development and evaluation of complex interventions were followed. Guidance on good practice for conducting feasibility studies and for conducting explanatory mixed methods were also followed. Several methods were employed to assess and ensure the study quality [37, 65]. It also provided new insights on methods to assess practitioners’ views.

The quantitative results showed an unexpectedly high level of satisfaction with the course and the content leading to the inclusion of specific questions in the semi-structured interviews to explore more deeply participants’ views on the biopsychosocial model in practice. In a main trial, the satisfaction survey could be sent a few weeks after completing the course in order to gather data on participants’ experience in implementing the content in clinical practice.

Whilst the intervention validity was carefully considered and its content informed by the scoping review results [19, 20], the validity of the scenarios would need to be considered: they were written by the researcher based on his clinical experience, and on theoretical aspects important for understanding pain mechanisms. Using an expert panel to assess their validity would be appropriate and exchanging the ones used for real-case scenarios that would be used to film professional actors or real patients, whilst considering ethical implications, could enhance their validity.

As there is not a clear-cut point when the integration of the BPS model started in Osteopathic Educational Institutions’ curricula, the inclusion criteria might have limited the recruitment rate. The recruitment rate was lower than expected (45 instead of 50): whilst this may not have a large impact on the findings on the feasibility of running a main trial, it is suggestive of a highly selective sample that was possibly keen on taking a course online.

Measuring knowledge could have been a useful outcome but there are no existing instruments assessing knowledge of the biopsychosocial model and the closest existing tools lack evidence regarding their reliability, e.g. the Pain Neurophysiology Questionnaire [51]. If additional questionnaires were used in a main trial, participants’ burden should be carefully considered. The purposeful absence of knowledge of the participants’ prior BPS training at recruitment could have confounded the results as groups may not have been successfully balanced by randomisation. However, to some extent this is mitigated by the similar baseline characteristics of both groups as measured by the PABS and ABS-MP. In a main trial, collection of prior exposure to BPS training after participant enrolment could effectively enable the assessment of BPS education at recruitment being unbalanced and confounding the results.

The breach of eligibility criteria (i.e. including two participants with 14 years of experience) would not be possible in a main trial and considerations about eligibility criteria is paramount.

The external validity of the findings on using e-learning as a form of CPD might be limited, as participants in the study did not pay to take the e-learning programme. Their satisfaction rating or acceptability of the intervention could have been different if a fee had been paid. The design of a main trial would need to include blinding of outcome assessors and data analysis and will need further consideration as a pragmatic trial comparing the effectiveness of the e-learning delivery compared to face-to-face delivery may provide insightful comparative data. This would require further feasibility testing. In order to use the e-learning programme in a main trial, updating the content would be required but it would be largely suitable. Recent surveys of osteopaths in the UK suggest that there is a need to enhance osteopaths’ BPS dispositions [3, 44]. Updating the e-learning programme is anticipated only to require a limited amount of work; therefore, the resources required in a main trial are expected to be similar to those required in this feasibility study.

## Conclusion

This mixed methods feasibility study supports that conducting an RCT would be feasible: the recruitment procedure, randomisation process and data collection were found feasible to use in a main trial. The sample was composed of experienced practitioners and the intervention was overall very well accepted. Using real scenarios or discussing the clinical scenarios with experts should be considered to improve the e-learning programme validity. The study followed recommendations on the conducting of mixed methods explanatory design and there were clear strategies implemented to ensure the quantitative and qualitative data quality.

## Supplementary Information

**Additional file 1.**

**Additional file 2.**

## Data Availability

Available as supporting evidence to the paper in two files

## Abbreviations

ABS-mp: Attitudes to Back pain Scale for musculoskeletal practitioners
LS: Limitations on sessions
PS: Psychology
CHS: Connection to Health care System
CC: Confidence and concern
RA: Reactivation
BM: Biomedical
BPS: Biopsychosocial
UCO: University College of Osteopathy
CI: Confidence interval
CPD: Continuous professional development
IQR: Interquartile range
IT: Information technology
LBP: Low back pain
MRC: Medical Research Council
NCOR: National Council for Osteopathic Research
NSLBP: Non-specific low back pain
PABS: Pain Attitudes and Beliefs Scale
PS: Psychosocial
RCT: Randomised controlled trial
SD: Standard deviation
